# Supplementary material for: Transient eco-evolutionary dynamics early in a phage epidemic have strong and lasting impact on the long-term evolution of bacterial defences
Source: PLoS Biol. 2023 Sep 15;21(9):e3002122. doi: 10.1371/journal.pbio.3002122 (PMC10530023; doi:10.1371/journal.pbio.3002122)
Supplement: S1 Text — (DOCX) [file pbio.3002122.s001.docx]

**Watson et al.**

**Transient eco-evolutionary dynamics early in a phage epidemic have strong and lasting impact on the long-term evolution of bacterial defenses**

**Supplementary Information**

**Transient selection for different types of resistance against bacteriophages**

1. **The model**

We want to model the transient evolution of a bacterial population exposed to phage predation. Initially, the bacteria population is fully susceptible ($S$ is the density of susceptible, or sensitive, bacteria) and is exposed to a lytic phage ($V$ is the density of free viral (phage) particles). Susceptible bacteria can either evolve surface resistance by mutation ($R$ is the density of surface resistant bacteria) or they can evolve CRISPR resistance after the acquisition of a new spacer targeting the phage ($C$ is the density of CRISPR resistant bacteria). Note that CRISPR resistant cells may also acquire surface resistance by mutation ($D$ is the density of cells that carry both surface resistance and CRISPR resistance) (Fig 1). The following dynamical system allows us to track the transient build-up of resistance via these two different mechanisms:

| $\frac{dS}{dt}=r\left( 1-\mu\right)S\left( 1-\frac{N}{K} \right)-aSV-mS+L C$  $\frac{dR}{dt}=r\mu S\left( 1-\frac{N}{K} \right)+rRe^{-c_{R}}\left( 1-\frac{N}{K} \right)-mR +L D$  $\frac{dC}{dt}=aSVA+rC\left( 1-\mu\right)e^{-\tau aV}\left( 1-\frac{N}{K} \right)-mC-L C$  $\frac{dD}{dt}=r\left( C\mu e^{-\tau aV}+De^{-c_{R}} \right)\left( 1-\frac{N}{K} \right)-mD-L D$  $\frac{dV}{dt}=aSV\left( 1-A \right)B-m_{v}V-a\left( S+C \right)V$ | (S1) |
| --- | --- |

where we assume that surface resistance carries a fitness cost $c_{R}$ while CRISPR resistance may suffer from a fitness cost $\tau$ when the CRISPR resistant cells are infected by the phage (this toxicity increases with the force of infection $aV$).

The total bacteria population size is defined as: $N=S+R+C+D$. The change in the total population size is given by:

| $\frac{dN}{dt}=\left( r\left( 1-\frac{N}{K} \right)\left( 1-\left( f_{R}+f_{D} \right)\left( 1-e^{-c_{R}} \right)-f_{C}\left( 1-e^{-\tau aV} \right) \right)-m-aV\left( 1-f_{R}-f_{C}-f_{D} \right)\left( 1-A \right) \right)N$ | (S2) |
| --- | --- |

where the frequencies of the different types of resistant cells are indicated as:

$$f_{R}=C/N$$

$$f_{C}=C/N$$

$$f_{D}=D/N$$

1. **Evolution of resistance**

Next, to understand the transient evolutionary dynamics of resistance, we focus on the dynamics of the frequencies of the different types of resistance.

**2.1 Evolution of surface resistance**

| $\frac{df_{R}}{dt}=\underset{mutation}{\underbrace{\mu rf_{S}\left( 1-\frac{N}{K} \right)}}+f_{R}\left( 1-f_{R} \right)\left( \underset{benefit}{\underbrace{aV\left( 1-A \right)}}-\underset{cost}{\underbrace{r\left( 1-e^{-c_{R}} \right)\left( 1-\frac{N}{K} \right)}} \right)-\underset{interference with C}{\underbrace{f_{R}f_{C}\left( aV\left( 1-A \right)-r\left( 1-e^{-\tau aV} \right)\left( 1-\frac{N}{K} \right) \right)}}-\underset{interference with D}{\underbrace{f_{R}f_{D}\left( aV\left( 1-A \right)-r\left( 1-e^{-c_{R}} \right)\left( 1-\frac{N}{K} \right) \right)+\underset{\begin{aligned} spacer \\ loss \end{aligned}}{\underbrace{Lf_{D}}}}}$ | (S3) |
| --- | --- |

The first term is the flux of mutation from susceptible bacteria with $f_{S}=S/N$.

The second term depends on the amount of genetic variation (i.e. $f_{R}\left( 1-f_{R} \right)$) and the strength of selection on surface resistance (the benefit minus the cost): (i) the benefit is to escape from phage predation, (ii) the cost depends on bacteria density because reproduction is density dependent.

The third term captures the potential interference with CRISPR resistance. It is usually negative (the presence of an alternative form of resistance reduces the competitive edge of this mutation) but this effect is reduced by the cost on CRISPR resistance.

The final two terms capture the potential interference with double resistant cells. It is usually negative (the presence of an alternative form of resistance reduces the competitive edge of this mutation) but this negative effect is reduced by the cost on CRISPR resistance, as well as the loss of spacer(s) from the double resistant cells.

**2.2 Evolution of CRISPR resistance**

| $\frac{df_{C}}{dt}=\underset{\begin{aligned} spacer \\ acquisition \end{aligned}}{\underbrace{aVAf_{S}}}-\underset{\begin{aligned} spacer \\ loss \end{aligned}}{\underbrace{Lf_{C}}} +f_{C}\left( 1-f_{C} \right)\left( \underset{benefit}{\underbrace{aV\left( 1-A \right)}}-\underset{cost}{\underbrace{r\left( 1-e^{-\tau aV} \right)\left( 1-\frac{N}{K} \right)}} \right)$  $\underset{interference with R}{\underbrace{-f_{C}f_{R}\left( aV\left( 1-A \right)-r\left( 1-e^{-c_{R}} \right)\left( 1-\frac{N}{K} \right) \right)}}\underset{interference with D}{\underbrace{-f_{R}f_{D}\left( aV\left( 1-A \right)-r\left( 1-e^{-c_{R}} \right)\left( 1-\frac{N}{K} \right) \right)}}$ | (S4) |
| --- | --- |
|  |  |

The first two terms correspond to the rate of spacer acquisition (influx of CRISPR resistance) and spacer loss (outflux of CRISPR resistance). In contrast to surface resistance, the influx of CRISPR resistance depends on viral density and does not depend on bacteria reproduction.

The second term depends on the amount of genetic variation (i.e. $f_{C}\left( 1-f_{C} \right)$) and the strength of selection on CRISPR resistance (the benefit minus the cost): (i) the benefit is to escape from phage predation, (ii) the cost depends on bacteria density because reproduction is density dependent but it also depends on phage density (because the cost of CRISPR resistance is only expressed when bacteria are infected).

The third term captures the interference with the surface resistance. It is usually negative (the presence of an alternative form of resistance reduces the competitive edge of this mutation) but this effect is reduced by the cost of surface resistance.

The fourth term captures the interference with the double resistance cells. It is usually negative (the presence of an alternative form of resistance reduces the competitive edge of this mutation) but this effect is reduced by the cost on surface resistance (double resistance cells do not suffer from the toxicity cost because the phage does not enter the cells).

**2.3 Evolution of double resistance**

| $\frac{df_{D}}{dt}=\underset{mutation}{\underbrace{\mu r\left( 1-e^{-\tau aV} \right)f_{C}\left( 1-\frac{N}{K} \right)}} -\underset{\begin{aligned} spacer \\ loss \end{aligned}}{\underbrace{Lf_{D}}}-f_{D}\left( 1-f_{D} \right)\left( \underset{benefit}{\underbrace{aV\left( 1-A \right)}}-\underset{cost}{\underbrace{r\left( 1-e^{-c_{R}} \right)\left( 1-\frac{N}{K} \right)}} \right)$  $\underset{interference with R}{\underbrace{-f_{D}f_{R}\left( aV\left( 1-A \right)-r\left( 1-e^{-c_{R}} \right)r\left( 1-\frac{N}{K} \right) \right)}}-\underset{interference with C}{\underbrace{f_{D}f_{C}\left( aV\left( 1-A \right)-r\left( 1-e^{-\tau aV} \right)\left( 1-\frac{N}{K} \right) \right)}}$ | (S5) |
| --- | --- |

The first term is the flux of mutation from CRISPR resistant bacteria that acquire a surface resistance.

The second term results from the loss of the CRISPR spacer(s) from the double resistant cells.

The third term captures the potential interference with surface resistant cells and depends on the amount of genetic variation (i.e. $f_{D}\left( 1-f_{D} \right)$) and the strength of selection on resistance (the benefit minus the cost): (i) the benefit is to escape from phage predation, (ii) the cost depends on bacteria density because reproduction is density dependent. It is usually negative (the presence of an alternative form of resistance reduces the competitive edge of this mutation) but this effect is reduced by the cost on surface resistance.

The fourth term captures the potential interference with CRISPR resistant cells. It is usually negative (the presence of an alternative form of resistance reduces the competitive edge of this mutation) but this negative effect is reduced by the cost on CRISPR resistance.

1. **Figures**

**S1** and **S2 Figs** show numerical simulations that mimic batch transfer experiments when one varies the initial doses of viruses and the carrying capacity of the bacterial populations. The comparison between (S1) and (S2) can be used to make specific predictions on the transient evolution of the different forms of resistance. The influx of surface resistance is governed by the mutation rate $\mu$ and the rate of reproduction. The influx of CRISPR resistance is governed by the acquisition rate $A$ and the density of viruses. Higher initial doses of free viruses are going to promote the acquisition of spacers and hence promote the evolution of CRISPR (versus surface resistance). Other parameters can also act on viral replication (like carrying capacity) and favour the evolution of CRISPR.

**S3 Fig** shows the outcome under different initial conditions to understand the effect of host replication on resistance evolution.

**S4 Fig** shows the transitory dynamics of the densities of the different types of cells and the density of free viruses, as well as the dynamics of the linkage disequilibrium between the two resistance loci. This linkage disequilibrium is always negative because the accumulation of different resistance mechanisms does not increase the amount of resistance (negative epistasis).
