# Supplementary figures and images for: Transient eco-evolutionary dynamics early in a phage epidemic have strong and lasting impact on the long-term evolution of bacterial defences
Source: PLoS Biol. 2023 Sep 15;21(9):e3002122. doi: 10.1371/journal.pbio.3002122 (PMC10530023; doi:10.1371/journal.pbio.3002122)

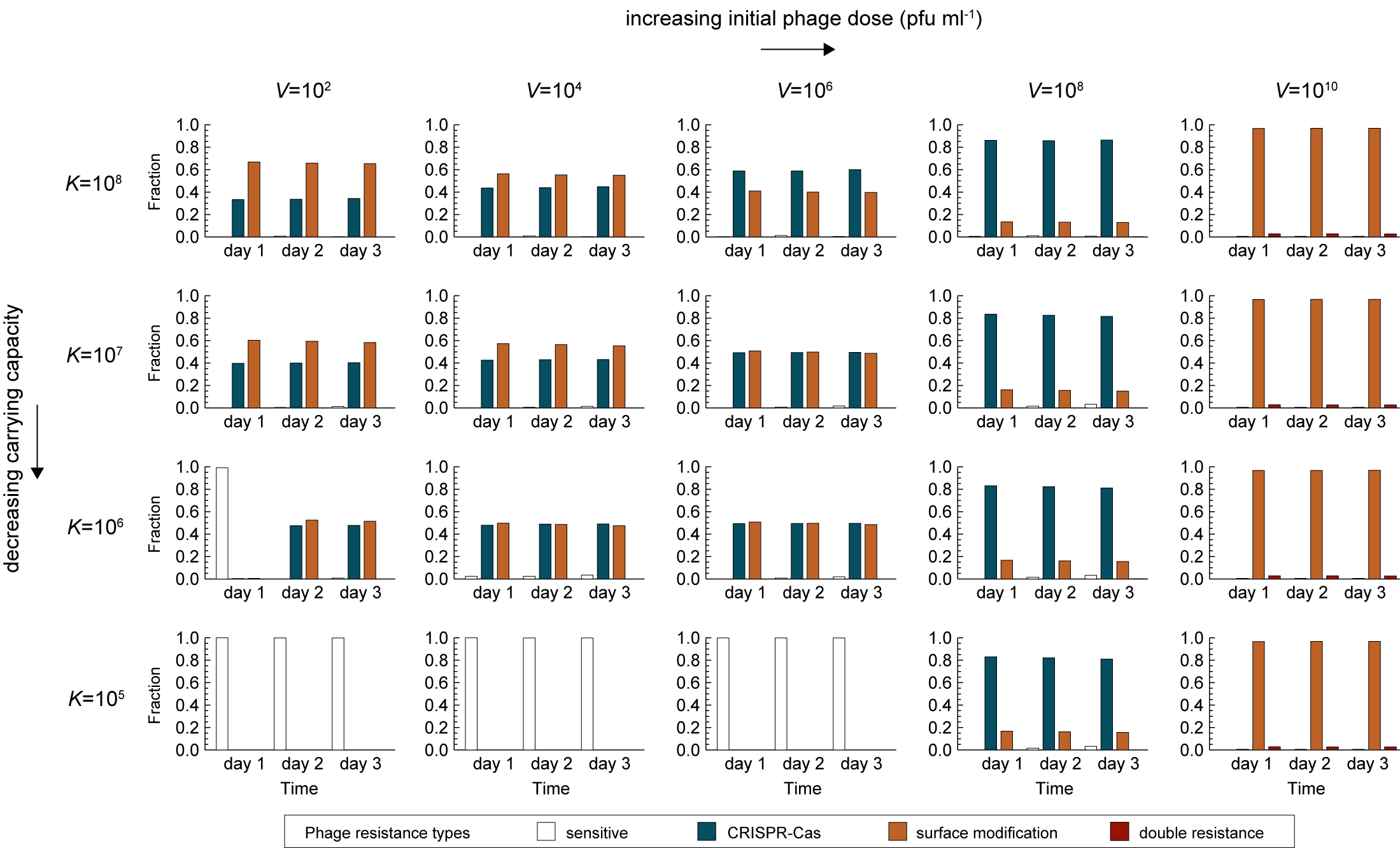

Supplement: S1 Fig — Frequency of the different bacterial resistance types (S (sensitive, white bars), C (CRISPR immune, blue), R (surface mutants, orange), or D (both CRISPR and surface mutants, red)) through time (3 days) and for different values of the initial doses of free viruses (V) and the carrying capacity (K). All the simulations started with an initial density of susceptible cells at K/100. Other parameter values: r = 1, m = 0, mv = 0, a = 10−8, B = 100, cR = 0.01, τ = 0.01, μ = 10−4, A = 5 10−4, L = 10−3. (TIF) [file pbio.3002122.s002.tif]

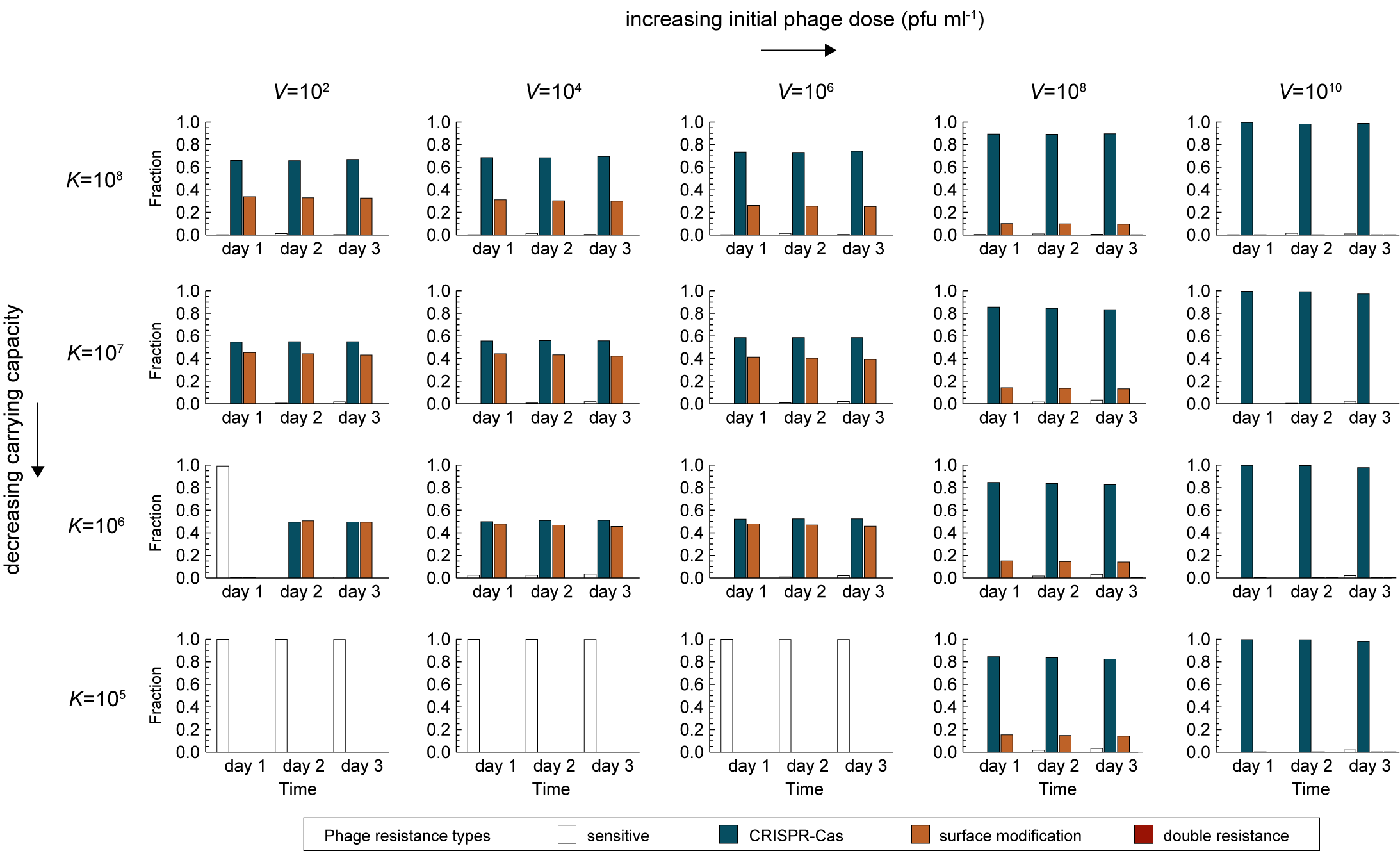

Supplement: S2 Fig — Frequency of the different bacterial resistance types (S (sensitive, white bars), C (CRISPR immune, blue), R (surface mutants, orange), or D (both CRISPR and surface mutants, red)) through time (3 days) and for different values of the initial doses of free viruses (V) and the carrying capacity (K), in the absence of an induced CRISPR immunity toxicity (τ = 0.0). All the simulations started with an initial density of susceptible cells at K/100. Other parameter values: r = 1, m = 0, mv = 0, a = 10−8, B = 100, cR = 0.01, τ = 0.0, μ = 10−4, A = 5 10−4, L = 10−3. (TIF) [file pbio.3002122.s003.tif]

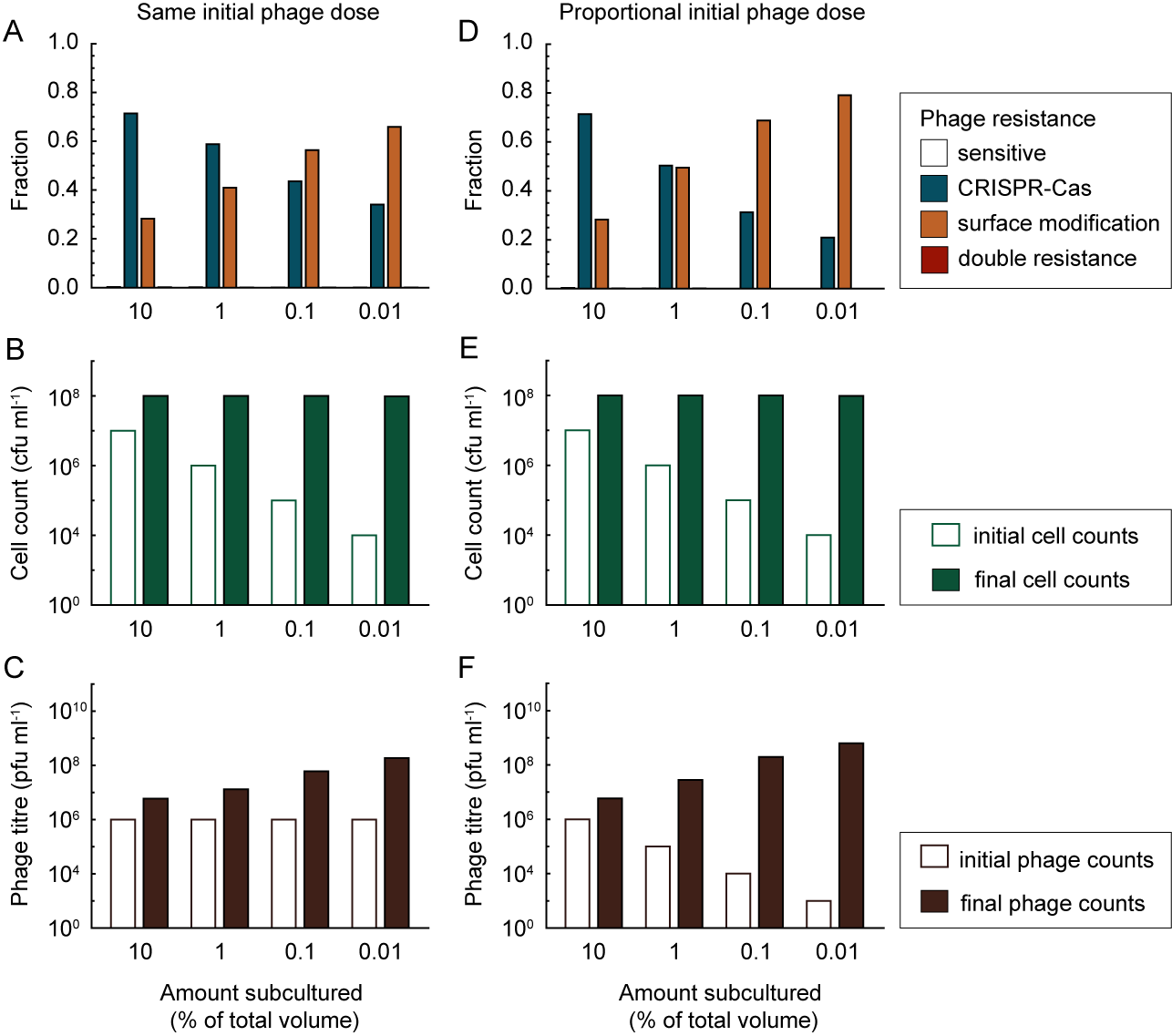

Supplement: S3 Fig — Plots show: (A, D) the predicted resistance type fractions, (B, E) cell counts, and (C, F) phage counts, with (A-C) the same initial phage dose (V = 106) or (D-F) when the initial dose of viruses was diluted to keep the same initial multiplicity of infection of 0.1. Amount of starting inoculum: 10% (K/10), 1% (K/100), 0.1% (K/1,000), 0.01% (K/10,000) of total final volume. Other parameter values: r = 1, m = 0, mv = 0, a = 10−8, B = 100, cR = 0.01, τ = 0.01, μ = 10−4, A = 5 10−4, L = 10−3. Panels (A-C) are also shown in Fig 2, alongside the experimental results (Fig 2D–2F). Data are available at https://doi.org/10.5281/zenodo.8193506. (TIF) [file pbio.3002122.s004.tif]

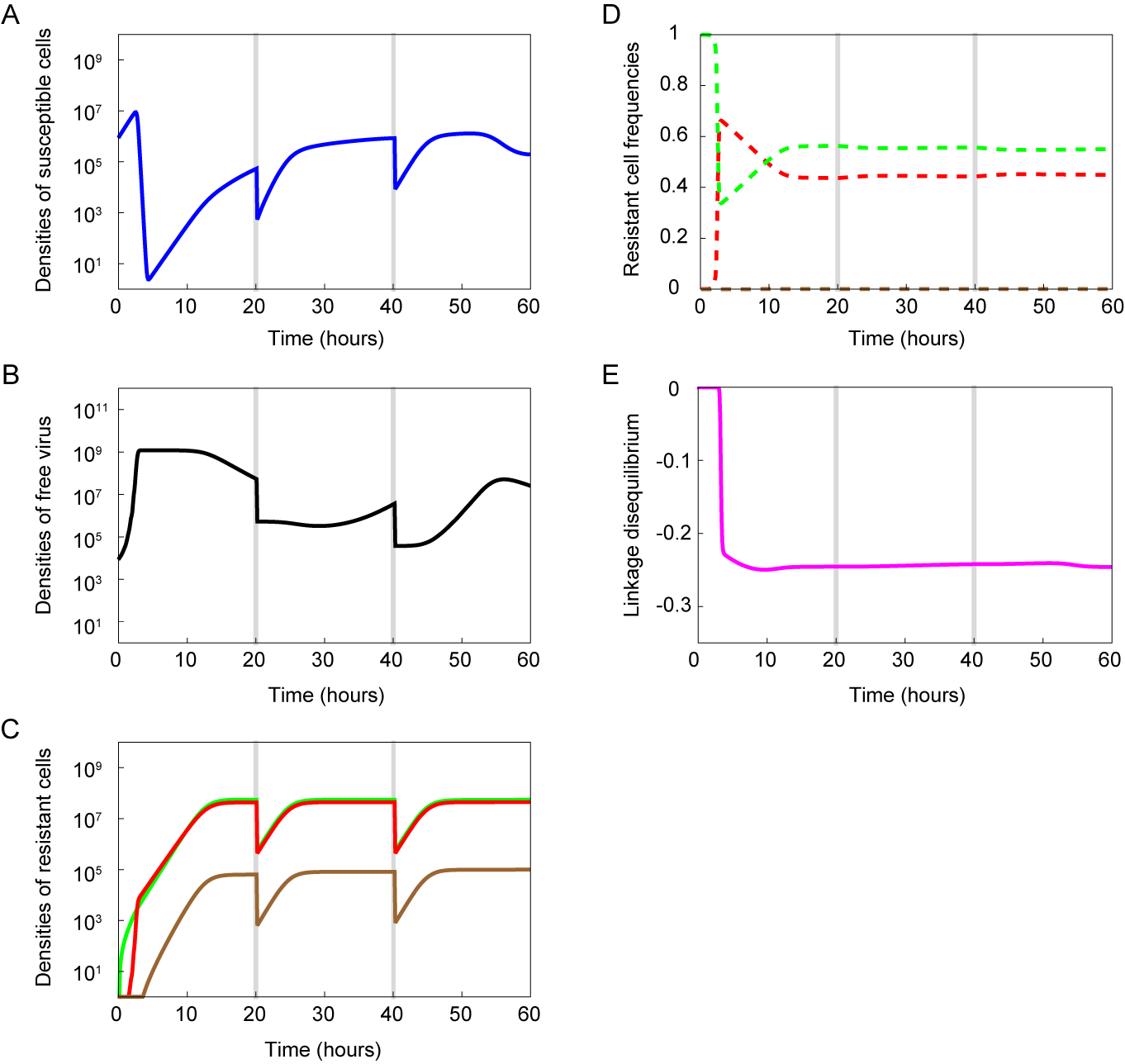

Supplement: S4 Fig — The plots show the dynamics for 3 transfers (20 hours between each transfer, indicated by a vertical grey line), including (A) density of S cells (susceptible, blue line), (B) density of V (viruses, black line), (C) densities of the different types of resistant cells (R (surface mutation, green line), C (CRISPR immune, red line), and D (both resistances, brown line), (D) frequencies of R, C, and D cells (dashed lines with same colours as in panel C), and (E) dynamics of linkage disequilibrium between the resistance loci across time. Linkage disequilibrium between the resistance loci is measured as: LD = fSfD−fRfC. Parameter values: r = 1, m = 0, mv = 0, a = 10−8, B = 100, cR = 0.01, τ = 0.01, μ = 10−4, A = 5 10−4, L = 10−3, K = 108, and (initial) V = 104. (TIF) [file pbio.3002122.s005.tif]

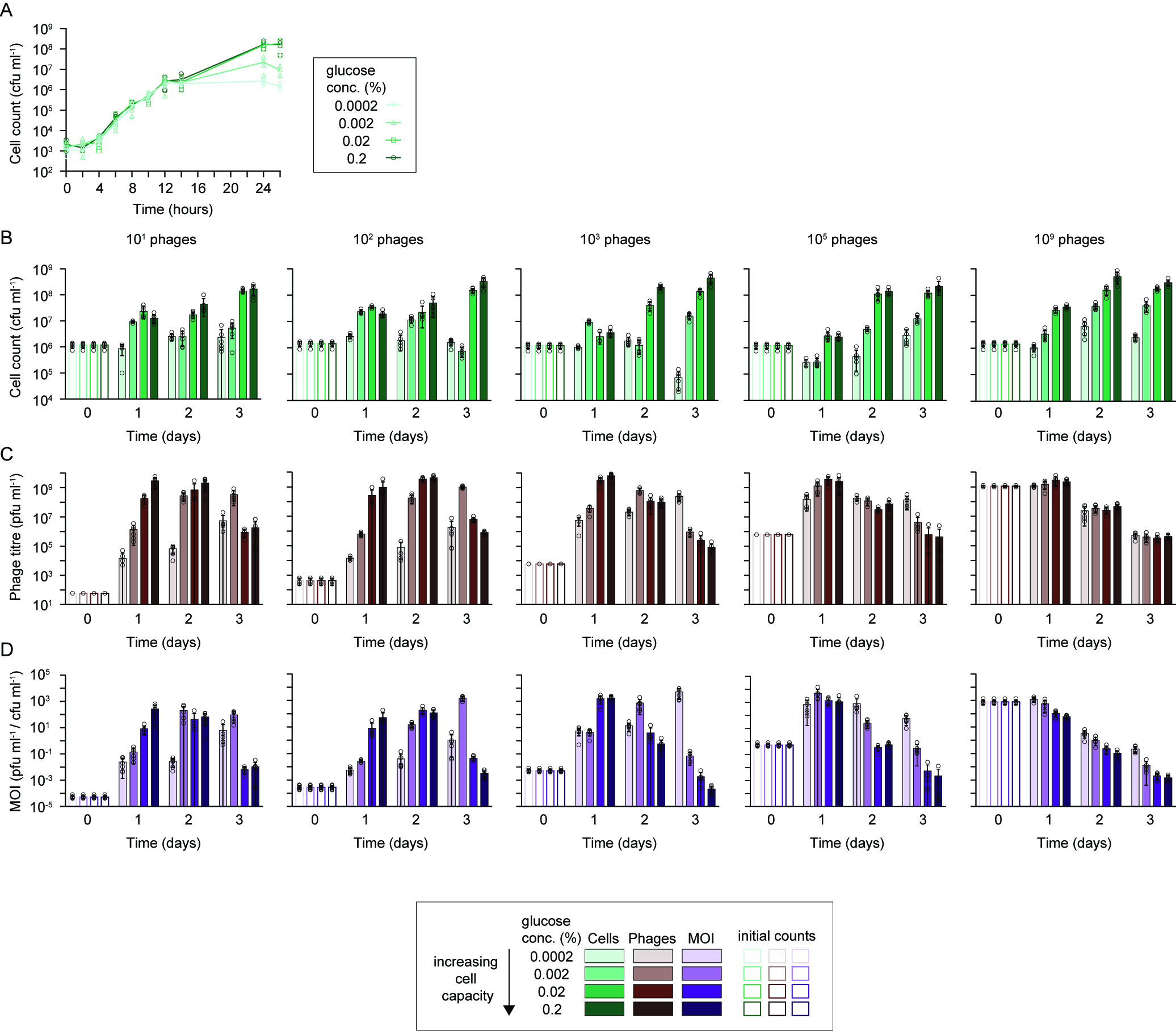

Supplement: S5 Fig — Plots show (A) growth curves of WT cultures grown in M9 containing different amounts of glucose (0.2%, 0.02%, 0.002%, and 0.0002%), (B) cell counts (CFU ml−1), (C) phage counts (PFU ml−1), and (D) the multiplicities of infection (MOI: phage count/cell count) across the 3-day experiment. Initial (day 0) values are depicted as white bars, increasing colour density (light-dark shades) represent increasing glucose concentrations in the growth media/culture carrying capacity. Data are available at https://doi.org/10.5281/zenodo.8193506. (TIF) [file pbio.3002122.s006.tif]

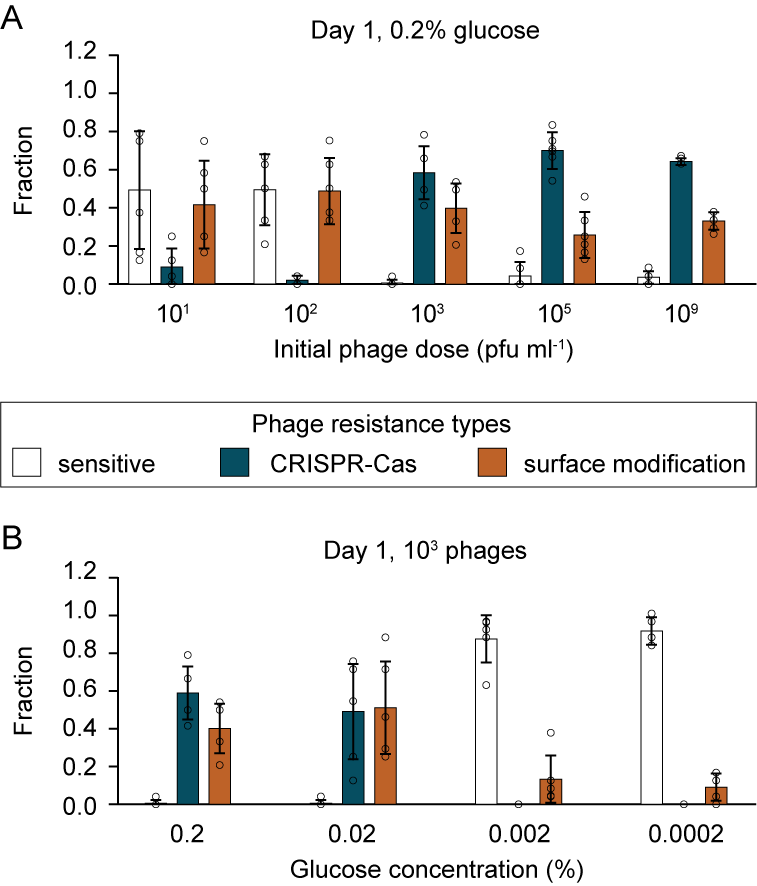

Supplement: S6 Fig — Fraction of each resistance type (white: phage sensitive, blue: CRISPR-Cas immune, orange: surface-based resistance) that evolved after 1 day of evolution following exposure of initially phage sensitive WT P. aeruginosa to (A) different amounts of DMS3vir phages (101, 103, 105, and 109 PFU ml−1) in media containing 0.2% glucose (highest carrying capacity) and (B) a moderate phage dose (103 PFU ml−1) in media containing different levels of glucose (0.2%, 0.02%, 0.002%, 0.0002% glucose, resulting in different carrying capacities; see S4A Fig). Data shown are the mean ± 1 standard deviation, 6 replicates per treatment, 24 clones tested per replicate. Data are available at https://doi.org/10.5281/zenodo.8193506. (TIF) [file pbio.3002122.s007.tif]
