# Supplementary material for: Transient eco-evolutionary dynamics early in a phage epidemic have strong and lasting impact on the long-term evolution of bacterial defences
Source: PLoS Biol. 2023 Sep 15;21(9):e3002122. doi: 10.1371/journal.pbio.3002122 (PMC10530023; doi:10.1371/journal.pbio.3002122)
Supplement: S1 Table — Fixed effects of glucose, cell density, phage density and initial phage inoculum, and treatment replicate as a random effect, are shown for each time point. Rows with selected models are shown in bold. “Phage” refers to initial phage inoculum size, while “log_phage” and “log_cell” indicate measured phage and cell densities, respectively. (DOCX) [file pbio.3002122.s008.docx]

| **(Intercept)** | **glucose** | **log_cell** | **log_phage** | **phage** | | **df** | **logLik** | | | **AICc** | | **delta** | | **weight** | |
| --- | --- | --- | --- | --- | --- | --- | --- | --- | --- | --- | --- | --- | --- | --- | --- |
| **T= 1-day post infection** | | | | |  | | |  |  | |  | |  | |  |
| **-31.4088** | **-** | **-** | **3.102901** | **0.364453** | | **4** | **-884.912** | | | **1777.837** | | **0** | | **0.38751** | |
| -30.4731 | 2.333508 | - | 2.966241 | 0.387754 | | 5 | -884.182 | | | 1778.385 | | 0.547847 | | 0.294659 | |
| -32.7203 | - | 0.218796 | 3.087841 | 0.363516 | | 5 | -884.555 | | | 1779.13 | | 1.293173 | | 0.20299 | |
| -31.1974 | 2.00583 | 0.100957 | 2.977234 | 0.383943 | | 6 | -884.12 | | | 1780.269 | | 2.43239 | | 0.114841 | |
| -34.0178 | - | - | 3.585071 | - | | 3 | -902.726 | | | 1811.46 | | 33.62259 | | 1.94E-08 | |
| -34.7215 | -2.15011 | - | 3.682213 | - | | 4 | -902.322 | | | 1812.657 | | 34.82042 | | 1.06E-08 | |
| -35.8714 | - | 0.259674 | 3.597894 | - | | 4 | -902.422 | | | 1812.859 | | 35.02156 | | 9.63E-09 | |
| -38.283 | -3.38692 | 0.439741 | 3.761776 | - | | 5 | -901.565 | | | 1813.151 | | 35.31414 | | 8.32E-09 | |
| -7.29194 | 16.11358 | - | - | 0.784458 | | 4 | -926.018 | | | 1860.05 | | 82.21321 | | 5.44E-19 | |
| -6.1196 | 16.51069 | -0.1785 | - | 0.783321 | | 5 | -925.928 | | | 1861.878 | | 84.04071 | | 2.18E-19 | |
| -11.0008 | - | 0.671885 | - | 0.803605 | | 4 | -940.374 | | | 1888.761 | | 110.9244 | | 3.17E-25 | |
| -6.51748 | - | - | - | 0.800267 | | 3 | -941.5 | | | 1889.007 | | 111.1705 | | 2.80E-25 | |
| -5.0236 | 20.21595 | - | - | - | | 3 | -961.545 | | | 1929.097 | | 151.2604 | | 5.53E-34 | |
| -4.10364 | 20.50837 | -0.13997 | - | - | | 4 | -961.522 | | | 1931.057 | | 153.2202 | | 2.07E-34 | |
| -3.65631 | - | - | - | - | | 2 | -971.966 | | | 1947.937 | | 170.1 | | 4.48E-38 | |
| -9.33632 | - | 0.841333 | - | - | | 3 | -971.098 | | | 1948.204 | | 170.3674 | | 3.92E-38 | |
| **T= 2-day post infection** | | | | |  | | |  |  | |  | |  | |  |
| -23.9903 | - | 1.450115 | 1.599456 | 0.149782 | | 5 | -1236.26 | | | 2482.543 | | 0 | | 0.49582 | |
| **-22.6156** | **-** | **1.582378** | **1.391144** | **-** | | **4** | **-1238.1** | | | **2484.206** | | **1.663467** | | **0.215828** | |
| -24.5147 | -0.9059 | 1.512292 | 1.617788 | 0.145486 | | 6 | -1236.22 | | | 2484.463 | | 1.919925 | | 0.189853 | |
| -23.7645 | -1.96563 | 1.70658 | 1.437049 | - | | 5 | -1237.88 | | | 2485.775 | | 3.232544 | | 0.098489 | |
| -14.6326 | 9.457741 | - | 1.586111 | 0.30066 | | 5 | -1247.02 | | | 2504.052 | | 21.50947 | | 1.06E-05 | |
| -16.4964 | - | - | 1.871859 | 0.330384 | | 4 | -1253.61 | | | 2515.243 | | 32.69987 | | 3.93E-08 | |
| -11.1312 | 10.01418 | - | 1.304623 | - | | 4 | -1254.02 | | | 2516.05 | | 33.50674 | | 2.63E-08 | |
| -12.6494 | - | - | 1.566861 | - | | 3 | -1260.9 | | | 2527.81 | | 45.26669 | | 7.34E-11 | |
| -11.5005 | 5.156819 | 1.522347 | - | - | | 4 | -1263.94 | | | 2535.884 | | 53.34131 | | 1.30E-12 | |
| -13.595 | - | 1.854544 | - | - | | 3 | -1265.03 | | | 2536.068 | | 53.5254 | | 1.18E-12 | |
| -13.7149 | - | 1.897715 | - | -0.04475 | | 4 | -1264.91 | | | 2537.827 | | 55.28439 | | 4.90E-13 | |
| -11.5447 | 5.075363 | 1.532788 | - | -0.00597 | | 5 | -1263.94 | | | 2537.896 | | 55.3529 | | 4.74E-13 | |
| -1.87957 | 15.67414 | - | - | 0.164593 | | 4 | -1271.51 | | | 2551.034 | | 68.49064 | | 6.65E-16 | |
| -1.18695 | 15.57846 | - | - | - | | 3 | -1273.33 | | | 2552.669 | | 70.12636 | | 2.93E-16 | |
| -1.04461 | - | - | - | 0.171518 | | 3 | -1285.01 | | | 2576.02 | | 93.47675 | | 2.50E-21 | |
| -0.33416 | - | - | - | - | | 2 | -1286.55 | | | 2577.096 | | 94.55273 | | 1.46E-21 | |
| **T= 3-day post infection** | | | | | | | |  |  | |  | |  | |  |
| **-16.995022** | **-** | **2.03555664** | **0.55147102** | **-0.191400** | | **5** | **-1274.6647** | | | **2559.35031** | | **0** | | **0.66632070** | |
| -17.339435 | -1.5271051 | 2.103227925 | 0.542990627 | -0.196125 | | 6 | -1274.4711 | | | 2560.971478 | | 1.621168373 | | 0.296245027 | |
| -10.17935 | - | 1.612735042 | - | -0.251981 | | 4 | -1279.5797 | | | 2567.173439 | | 7.823129504 | | 0.013332522 | |
| -20.02456 | - | 2.158428152 | 0.760673763 | - | | 4 | -1279.6290 | | | 2567.272004 | | 7.921694212 | | 0.012691392 | |
| -10.768006 | -2.0079994 | 1.70988591 | - | -0.256854 | | 5 | -1279.2650 | | | 2568.550983 | | 9.200673352 | | 0.006695492 | |
| -20.133321 | -0.3961623 | 2.176817493 | 0.75977039 | - | | 5 | -1279.6173 | | | 2569.255605 | | 9.90529541 | | 0.004707342 | |
| -10.75746 | - | 1.5459523 | - | - | | 3 | -1288.3825 | | | 2582.773391 | | 23.42308112 | | 5.46E-06 | |
| -10.950153 | -0.64100947 | 1.57673391 | - | - | | 4 | -1288.3559 | | | 2584.725752 | | 25.37544172 | | 2.06E-06 | |
| 3.048364661 | 10.2602745 | - | -0.33813037 | -0.203665 | | 5 | -1307.7367 | | | 2625.494291 | | 66.14398116 | | 2.89E-15 | |
| 0.590754189 | 12.62890254 | - | - | -0.151574 | | 4 | -1309.2742 | | | 2626.562503 | | 67.21219267 | | 1.69E-15 | |
| -0.0436170 | 12.64090345 | - | - | - | | 3 | -1311.3255 | | | 2628.659407 | | 69.30909746 | | 5.93E-16 | |
| 0.963400705 | 11.57801632 | - | -0.15204647 | - | | 4 | -1310.9919 | | | 2629.997816 | | 70.64750614 | | 3.04E-16 | |
| 5.773130979 | - | - | -0.65828154 | -0.246481 | | 4 | -1314.1221 | | | 2636.258252 | | 76.90794214 | | 1.33E-17 | |
| 3.635925332 | - | - | -0.48025385 | - | | 3 | -1318.5842 | | | 2643.176849 | | 83.82653942 | | 4.18E-19 | |
| 1.231046881 | - | - | - | -0.143057 | | 3 | -1320.6772 | | | 2647.362764 | | 88.01245429 | | 5.15E-20 | |
| 0.637355231 | - | - | - | - | | 2 | -1322.2186 | | | 2648.441465 | | 89.09115502 | | 3.00E-20 | |

**S1 Table: AIC selection tables for binomial generalised linear mixed effects models.** Fixed effects of glucose, cell density, phage density and initial phage inoculum, and treatment replicate as a random effect, are shown for each timepoint. Rows with selected models are shown in bold. ‘Phage’ refers to initial phage inoculum size, whilst ‘log_phage’ and ‘log_cell’ indicate measured phage and cell densities, respectively.
