# Supplementary material for: Transient eco-evolutionary dynamics early in a phage epidemic have strong and lasting impact on the long-term evolution of bacterial defences
Source: PLoS Biol. 2023 Sep 15;21(9):e3002122. doi: 10.1371/journal.pbio.3002122 (PMC10530023; doi:10.1371/journal.pbio.3002122)
Supplement: S2 Table — “Phage” refers to initial phage inoculum size, while “log_phage” and “log_cell” indicate measured phage and cell densities, respectively, at each time point. (DOCX) [file pbio.3002122.s009.docx]

|  | **Estimate** | **Std. Error** | **z value** | **Pr(>\|z\|)** |
| --- | --- | --- | --- | --- |
| **T= 1-day post infection** | |  |  |  |
| **(Intercept)** | -31.4092 | 3.34962 | -9.37694 | 6.79E-21 |
| **Phage** | 0.364452 | 0.056302 | 6.473196 | 9.60E-11 |
| **log_phage** | 3.102939 | 0.357563 | 8.678031 | 4.03E-18 |
| **T= 2-day post infection** | |  |  |  |
| **(Intercept)** | -22.6156 | 2.433671 | -9.29281 | 1.50E-20 |
| **log_cell** | 1.582378 | 0.225369 | 7.021265 | 2.20E-12 |
| **log_phage** | 1.391144 | 0.20461 | 6.799011 | 1.05E-11 |
| **T= 3-day post infection** | |  |  |  |
| **(Intercept)** | -16.995 | 2.579733 | -6.58789 | 4.46E-11 |
| **Phage** | -0.1914 | 0.058546 | -3.26924 | 0.001078 |
| **log_cell** | 2.035555 | 0.220627 | 9.226229 | 2.80E-20 |

**S2 Table: Estimates for binomial generalised linear mixed effects models with variables that were retained following AIC selection.** ‘Phage’ refers to initial phage inoculum size, whilst ‘log_phage’ and ‘log_cell’ indicate measured phage and cell densities, respectively, at each timepoint.
