# Supplementary material for: Transient eco-evolutionary dynamics early in a phage epidemic have strong and lasting impact on the long-term evolution of bacterial defences
Source: PLoS Biol. 2023 Sep 15;21(9):e3002122. doi: 10.1371/journal.pbio.3002122 (PMC10530023; doi:10.1371/journal.pbio.3002122)
Supplement: Attachment — Submitted filename: Watson_et_al_Response_to_Reviewers.pdf [file pbio.3002122.s010.pdf]

We thank the Reviewers and Editor for their helpful comments and suggestions. Responses to the comments are below.

The manuscript title has been changed as suggested by the Editorial team, as is now 'Transient eco-evolutionary dynamics early in a phage epidemic have strong and lasting impact on the long-term evolution of bacterial defenses'

Reviewer #1

The Reviewer points out that that the model is original, but the approach is standard, so claims of it being 'novel' should be avoided. The word 'novel' has been removed from the text.

The Reviewer suggests building on the discussion to place our work in a more general context. In response, we have extensively expanded the discussion to include other models of CRISPR evolution, incorporating the suggested citations and others.

Reviewer #2

Reviewer also suggests adding to the discussion, to comment on: 1) constitutive and inducible systems, in particular the ongoing cost of expressing CRISPR systems, and 2) how the different defenses affect the fitness of individuals, as well as the community. Text has been added to discuss these ideas.

Academic Editor

The editor pointed out that it would be useful to expand on how 1) the initial population sizes and the resulting mutation supply, and 2) the reduced cell density, or 'sparseness', resulting from low glucose concentrations affected the resistance types observed. Text has been added to the manuscript to address these points.

The typos have been corrected, as suggested.

Data and code have been deposited in Zenodo: https://doi.org/10.5281/zenodo.8193506
